# Effect of Community-Based Occupational Therapy on Health-Related Quality of Life and Engagement in Meaningful Activities of Women with Breast Cancer

**DOI:** 10.1155/2018/6798697

**Published:** 2018-04-17

**Authors:** Daiva Petruseviciene, Deive Surmaitiene, Daiva Baltaduoniene, Egle Lendraitiene

**Affiliations:** ^1^Department of Rehabilitation, Medical Academy, Lithuanian University of Health Sciences, Kaunas, Lithuania; ^2^Kulautuva Hospital of Rehabilitation, Hospital of Lithuanian University of Health Sciences Kauno Klinikos, Kaunas, Lithuania

## Abstract

We aimed to evaluate the short-term effects of community-based occupational therapy on health-related quality of life and engagement in meaningful activities among women with breast cancer. An open label randomized controlled trial study design was applied. The participants were members of various societies of women with cancer. In total, 22 women have participated in the study. Participants of the experimental group (*n* = 11) participated in a 6-week community-based occupational therapy program and the usual activities of various societies, whereas the control group (*n* = 11) women participated in the usual activities of the societies only. 1 of the participants withdrew during the course; therefore 21 completed the study successfully. Participants of both groups were assessed for health-related quality of life and the participants of the experimental group were assessed for engagement in meaningful activities. The evaluation was carried out during the nonacute period of the disease—at the beginning of the study and after 6 weeks. Women of the experimental group demonstrated statistically significantly better scores in the global quality of life, role functions, physical, emotional, cognitive, and social functions, fatigue, insomnia, financial impact, systemic therapy side effects, and breast symptoms scales compared to the control group participants (*p* < 0.05) after the 6 weeks, as measured by the* EORTC QLQ-C30* questionnaire and its breast cancer module* QLQ-BR23*. Furthermore, women of the experimental group demonstrated significant greater engagement in meaningful activities when applying community-based occupational therapy (*p* < 0.05), as measured by using the Engagement in Meaningful Activities Survey (EMAS). The evaluation of the associations between the women's engagement in meaningful activities and changes in health-related quality of life showed that greater engagement in meaningful activities was associated with better emotional functions and a lower level of insomnia (*p* < 0.05). Based on the results of our study, we recommend applying occupational therapy in the field of community healthcare in order to maintain or improve breast cancer patients' health-related quality of life and suggest involving women into meaningful activities during community-based occupational therapy after clarifying which activities are important to them.

## 1. Introduction

On the global scale, cancer causes one-seventh of all deaths. According to the most recent estimates, there have been over 14 million new cases of cancer and over 8 million deaths from cancer registered worldwide, and it is expected that, due to the increasing numbers and aging of the population, these numbers will increase to, accordingly, 21.7 million and 13 million by the year 2030 [1].

Breast cancer, defined by the National Cancer Institute as cancer forming in breast tissues, is the most common malignant tumor among women and in as many as 19 out of 21 regions it is the leading cause of death among women in both the developed and the developing countries [2–4]. Over 1.5 million new cases of invasive breast cancer are registered globally every year, and this is the single most common form of cancer among women that is prevalent in absolutely all regions of the world [2, 5]. In European countries, breast cancer is also the most frequently diagnosed malignant tumor in women, and the prevalence of this disease has been increasing in most countries over the last decades [3, 6]. Despite the continuous improvement in the diagnostic and treatment techniques, breast cancer remains one of the most common cancers among women in Lithuania as well as in the rest of the world (in Lithuania, breast cancer is currently the second most common cancer after skin cancer (melanoma) and is undoubtedly their most urgent health problem). Even though survival rates in Lithuania are among the lowest, the overall situation is improving: breast cancer is detected at increasingly earlier stages, and women's survival is improving [7].

Unfortunately, the completion of breast cancer treatment does not mean the end of the disease [8]. Healthcare institutions with all the necessary services and specialists ensure integrated assistance to breast cancer patients. However, after discharge, women frequently experience emotional stress because of short-term or long-term adverse effects of the therapy, the risk of recurrence, difficulty getting information about their health status and its possible changes, financial problems, difficulty getting back to work, impaired sexual life, and so forth. Improving survival rates and the risk of late-onset adverse reactions increase the need for healthcare and rehabilitation services on the community level [3, 9, 10]. Consequently, quality of life questions related to the financial impact of the disease, concern about the future of the family, fear of disease recurrence, insufficient preparation for possible late-onset adverse reactions to the treatment, and possible changes in social support become increasingly relevant [11].

Today, it is the field of health-related quality of life that is being most actively developed in Lithuania as well as abroad [12]. Over the last several decades, breast cancer treatment has become more effective, which resulted in a significant increase in patients' long-term survival. Schoormans et al. (2015) stated that because of this, researchers had to focus increasingly on long-term consequences of the disease or the treatment, which they related to the quality of life, thus developing the concept of breast cancer patients' health-related quality of life [13].

The impact of the diagnosis and treatment of breast cancer differs depending on the patient, resulting in varying numbers and degrees of physical and psychological sequelae. The consequences may significantly impair a woman's ability to participate in meaningful activities, including housework, return to work, and adequate performance of the main social roles and responsibilities. This, in turn, may negatively affect the economic status and disrupt interpersonal relations, thus further impairing the woman's engagement in meaningful activities as well as her psychoemotional status and the quality of life. Despite the fact that the combined effect of breast cancer and its treatment may manifest themselves at any moment and last for a very long time, it is essential for the patients to resume their daily roles as soon as possible after the completion of the treatment and this is where the role of the occupational therapist is especially important [14]. According to Pergolotti et al. (2016), the economic effectiveness of occupational therapy and its usefulness in reducing disease consequences have already been proven, and yet occupational therapy is still rarely applied in the rehabilitation of cancer patients [15].

The theory of occupational therapy states that human subsistence is a social process, and people need meaningful activity; its absence is a major threat to human health, and thus occupational therapists in their professional practice strive to achieve their patients' maximum capacity to increase their ability to engage in activities that they see as important and meaningful [16, 17]. According to Cook and Thompson (2015), the meaningfulness of life significantly affects a person's physical, mental, and social wellbeing. Engagement in purposeful activities contributes to the satisfaction of the need for the meaningfulness of life, but only if this activity motivates the person, is interesting, can be successfully completed, and helps to achieve other significant aims. Engagement in purposeful activities directly affects a person's emotional condition, self-esteem, and self-confidence. Purposeful activities have been recognized as directly affecting the quality of life, and thus an important task when working with a patient is to clarify which activities are important for him or her. When purposeful activity is selected in accordance with the person's interests and priorities, it becomes meaningful [18]. Occupational therapists strive to and can help cancer patients live a meaningful life, be self-sufficient, participate in activities, be able to manage themselves and others, engage in work, leisure, and health promotion activities, and be a part of social groups and the family [16]. Occupational therapists' holistic approach and creative decisions may improve breast cancer patients' cognitive and physical capacity, mental health, and the quality of life [15].

When working in communities, occupational therapists have to be familiar with the characteristics of community-based activity in order to be able to pursue meaningful activities based on the community members' priorities. Active participation in community activities, social support, and engagement in meaningful activities positively affect community members' health and the quality of life [19]. Based on this theory, we wanted to determine the influence of community-based occupational therapy on breast cancer patients' health-related quality of life and engagement in meaningful activities.

No similar research was found and we were the first in Lithuania who made a study analyzing changes in health-related quality of life, engagement in meaningful activities, and its associations among breast cancer patients when applying occupational therapy in the field of community healthcare. We hope that the results of our study will be useful for occupational therapists working with breast cancer patients' communities.

Consequently, all these reasons have affected the following aim of the study: to evaluate the short-term effects of community-based occupational therapy on health-related quality of life and engagement in meaningful activities among women with breast cancer.

## 2. Methods

This randomized controlled trial was conducted with the permission of the Bioethics Center of the Lithuanian University of Health Sciences (number BEC-SR(M)-186), the permission of the European Organization for Research and Treatment of Cancer (EORTC) Quality of Life Group to use the quality of life questionnaire EORTC QLQ-C30 and its module EORTC QLQ-BR23 in the study, and the permission of the Associate Professor A. M. Eakman from Colorado State University to use the Engagement in Meaningful Activities Survey (EMAS) in the study. The study was performed during May–June 2016 in Kaunas city, with the mediation of the chairwomen of two societies of women who either had overcome their breast cancer or were still fighting it on the community level.

Eligible women were adult (≥18 years of age) and Lithuanian-speaking, with diagnosed breast cancer and functional status being ≤2b according to the ECOG* (Eastern Cooperative Oncology Group)* scale of performance status, were able to understand the essence of the study and simple instructions, were familiarized with the aim of the study, and volunteered to participate. Exclusion criteria were the following: refusal to participate in the study, women with severe decompensated comorbidities and mental diseases, and being incapable of independently filling out the questionnaires, and whose functional status was >2b according to the ECOG scale of performance status.

All the participants who met the inclusion criteria were in advance familiarized with the study, its aims, objectives, and methods, the confidentiality of the data, and the possibility of withdrawing from the study at any time and provided a written consent to participate in the study.

In total, 22 members of breast cancer patients' societies met the inclusion criteria. All participants had been diagnosed with breast cancer (code C50 according to the ICD-10-AM). The participants were randomly distributed into two groups: the experimental group and the control group, following simple randomization procedure (computerized random numbers). The distribution of the participants was equal in both groups. The participants were also distributed into two groups based on the analyzed characteristics: age, employment status, the duration of the disease, the stage of the disease, the location of the tumor, metastases, and functional status according to the ECOG. Detailed characteristics of the participants are presented in Table 1.

There were no significant differences in the participants' distribution between the groups concerning their age (*χ*^2^(1) = 0, *p* = 1), employment status (*p* = 1), the duration of the disease (*χ*^2^(1) = 0, *p* = 1), the stage of the disease (*p* = 0.311), the location of the tumor (*χ*^2^(1) = 0, *p* = 1), metastases (*p* = 1), or functional status according to the ECOG (*p* = 1), *p* > 0.05.

During the study, we evaluated changes in women health-related quality of life and experimental group participants' engagement in meaningful activities during the nonacute period of the disease. The methods used in the study were the following: a survey using a questionnaire designed by the author, testing, experiment, and statistical data analysis. During the survey, we evaluated the participants' sociodemographic data (age and employment status) and clinical factors: the duration of the disease, the stage of the disease, the location of the tumor, metastases, and functional status according to the ECOG. Testing was conducted twice: at the beginning of the study and after 6 weeks. In order to evaluate changes in the participants' health-related quality of life, we used the EORTC QLQ-C30 questionnaire and its module QLQ-BR23. The principal questionnaire, EORTC QLQ-C30, consists of 30 questions forming the global health status and quality of life scale, five functional scales, three symptom scales, and six separately evaluated components. The EORTC module QLQ-BR23 is a more specific questionnaire designed for breast cancer patients and consisting of 23 questions forming four functional scales and four symptom scales. After the calculation of the results according to the formulas presented by the authors, the score intervals in all scales and separately evaluated components ranged from 0 to 100 points. The questionnaires have been translated and adapted in many languages including Lithuanian and are suitable for the evaluation of cancer patients' health-related quality of life. In Lithuania, Banienė and Šinkariova (2015) evaluated the internal consistency of the scales of those questionnaires (0.6 ≤ *α* ≤ 0.9) and, based on the obtained results, stated that the questionnaires are suitable for studies of breast cancer patients [20].

The testing of the experimental group participants' engagement in meaningful activities was also conducted at baseline and at the end of the study, using the Engagement in Meaningful Activities Survey. This survey has not been translated into Lithuanian and has not been adapted in Lithuania. For this reason, studies for the evaluation of the reliability and validity of the survey should be conducted. The results of the survey on engagement in meaningful activities reflect the level of the subjects' engagement in meaningful activities. The survey consists of 12 statements, each of which has the minimal value of 1 point and the maximal value of 4 points, where 1 point means “rarely”; 2 points, sometimes; 3 points, often; and 4 points, always. Engagement in meaningful activities was evaluated by a sum score. There is a growing body of scientific literature validating the psychometric properties of this test. The association of the results of this test with those of the evaluation of satisfaction with life and health-related quality of life has already been proven. Eakman with coauthors (2010) have proven the test-retest reliability of this instrument (*r* = 0.56) and evaluated the internal consistency of the scale (*α* = 0.89), using the English version of this test in adult population [21]. All study participants filled out the questionnaires independently.

During the course of the study, the women participated in a 6-week community-based occupational therapy program and the usual activities of various societies, whereas the control group women participated in the usual activities of the societies only.

The community-based occupational therapy program was designed taking into account the most common sequelae of breast cancer and its treatment identified in literature: arm swelling, reduced range of motion of the arm, fatigue, and impaired quality of life. According to Scaffa and Reitz (2014), active participation in community activities, social support, and engagement in meaningful activities have a positive effect on community members' health and quality of life [19]. Cook and Thompson (2015) supported this opinion stating that meaningful activities improve the quality of life [18]. For this reason, by applying this program, we strived to improve not only the experimental group participants' health-related quality of life, but also their engagement in meaningful activities.

During the study, we applied occupational therapy in groups. During each session, purposeful activities were combined with creative techniques, and the sessions were made meaningful through voluntary assistance to others, that is, emotional help when communicating with other women who had the same problems and so forth. The experimental group participants also received individual consultations by an occupational therapist if needed.

Duration of the program consisted of six meetings. The description of the program is provided in Table 2.

Occupational therapy sessions in groups were organized four times: once weekly at an arranged time in the evening (after work), up to 1.5 hours/day.

Note that the sessions in occupational therapy groups took place in a cozy and comfortable environment, and all the tools and materials required for the activities were supplied and were of good quality. Prior to the sessions, the participants received all the necessary information, and the focus during the sessions was on the process rather than on the results.

Statistical data analysis was conducted using the* IBM SPSS Statistics 22* software. The data were described by using the standard numeral characteristics (min and max values, median, and mean). The distributions were compared by applying the nonparametric criteria. Two dependent samples before and after the intervention were compared by applying the nonparametric Wilcoxon criterion and two independent samples by using the Mann–Whitney-Wilcoxon criterion. In order to determine the presence of a relationship between engagement in meaningful activities and health-related quality of life, we calculated Spearman's correlation coefficient. The difference or the relationship between the indices were considered to be statistically significant when *p* < 0.05.

## 3. Results

After six weeks, of 22 women randomized into the study, 21 completed the trial. The experimental group consisted of 10 participants and the control group consisted of 11. One woman withdrew from the study because of breast cancer recurrence. During the study, when applying occupational therapy in the community, we strove to improve the participants' engagement in meaningful activities and thus their health-related quality of life. For this reason, the women's active participation in the assignments was highly important for us, and thus, to determine the actual impact of the applied program on the aforementioned indices, we only calculated the results for women who were involved in the study.

The health-related quality of life of women with breast cancer was evaluated during the nonacute period of the disease by calculating the scores in separate scales of the EORTC QLQ-C30 questionnaire and its module QLQ-BR23. The score could range from 0 to 100 points. A higher score in the global quality of life scale indicated better quality of life, a higher score in the functional scales better functioning, and a higher score in the symptom scales a greater degree of the manifestation of the symptom.

The evaluation of the participants' health-related quality of life at baseline using the EORTC QLQ-C30 questionnaire and its module QLQ-BR23 showed that the groups were homogeneous, *p* > 0.05 (Table 3).

Prior to the application of the community-based occupational therapy, the poorest evaluations in the EORTC QLQ-C30 questionnaire among the experimental group participants were on the global quality of life (50 (33.33–83.33; 50.75)), emotional functions (50 (8.33–100; 52.27)), cognitive functions (50 (0–83.33; 54.55)), and social functions (50 (16.67–83.33; 48.49)). The participants were also found to demonstrate the highest degrees of the manifestation of insomnia (66.67 (0–100; 66.67)) and the financial difficulties (66.67 (33.33–100; 69.7)). The results of the EORTC QLQ-BR23 questionnaire showed that the participants gave the poorest evaluations of the future perspective (33.33 (0–66.67; 33.33)) and demonstrated the highest degrees of the manifestation of breast symptoms (41.67 (8.33–75; 38.64)) such as pain, edema, tenderness, or skin problems.

Among the control group participants, the poorest evaluations in the EORTC QLQ-C30 questionnaire at baseline were on the global quality of life (58.33 (33.33–91.67; 59.85)), and the participants were found to demonstrate the highest degrees of the manifestation of insomnia (66.67 (0–66.67; 48.49)) and financial impact (66.67 (0–100; 51.52)). The results of the EORTC QLQ-BR23 questionnaire showed that the participants gave the poorest evaluations of the future perspective (33.33 (0–66.67; 27.27)) and demonstrated the highest degrees of the manifestation of arm symptoms (44.44 (0–88.89; 38.38)) such as pain and edema.

The evaluation of changes in the overall scores of the quality of life questionnaire EORTC QLQ-C30 scales between the groups showed that, compared to baseline data, after six weeks (at the end of the study), the experimental group demonstrated statistically significantly better results in the global quality of life (*U* = 0; *p* = 0.001; *r* = 0.85), physical functions (*U* = 20, *p* = 0.013; *r* = 0.54), role functions (*U* = 25.5; *p* = 0.036; *r* = 0.46), emotional functions (*U* = 8.5; *p* = 0.001; *r* = 0.72), cognitive functions (*U* = 10; *p* = 0.001; *r* = 0.72), social functions (*U* = 13; *p* = 0.002; *r* = 0.66), fatigue (*U* = 5; *p* = 0.001; *r* = 0.78), insomnia (*U* = 19; *p* = 0.01; *r* = 0.59), and financial impact (*U* = 23.5; *p* = 0.024; *r* = 0.52) scales, *p* < 0.05. The scores in other functional or symptom scales of the quality of life did not differ statistically significantly between the groups,* p* ≥ 0.05 (Table 4).

The evaluation of changes in the overall scores in the scales of the quality of life questionnaire module EORTC QLQ-BR23 between the groups showed that, compared to baseline data, after six weeks (at the end of the study), the experimental group demonstrated statistically significantly better results in the systemic therapy side effects (*U* = 11; *p* = 0.001; *r* = 0.68) and breast symptoms (*U* = 24.5; *p* = 0.029; *r* = 0.48) scales, *p* < 0.05. The scores in other functional or symptom scales did not differ statistically significantly between the groups,* p* ≥ 0.05 (Table 5).

Considering the calculated strength of the effect, when a moderate effect was ≥0.5 and a strong effect was ≥0.8, we found not only statistically, but also clinically significant changes (*p* < 0.05) in the scores of the global quality of life (0.85), physical (0.54), emotional (0.72), cognitive (0.72), and social (0.66) functions, fatigue (0.78), insomnia (0.59), financial impact (0.52), and systemic therapy side effects (0.68) scales.

Among the experimental group women with breast cancer, engagement in meaningful activities was evaluated during the nonacute period of the disease. The evaluation was performed based on the results of the Engagement in Meaningful Activities Survey (EMAS)—the sum score that could range from 12 to 48 points, where <29 points indicated insufficient engagement; 29–41 points, moderate engagement; and >41 points, good engagement.

**Figure 1:**
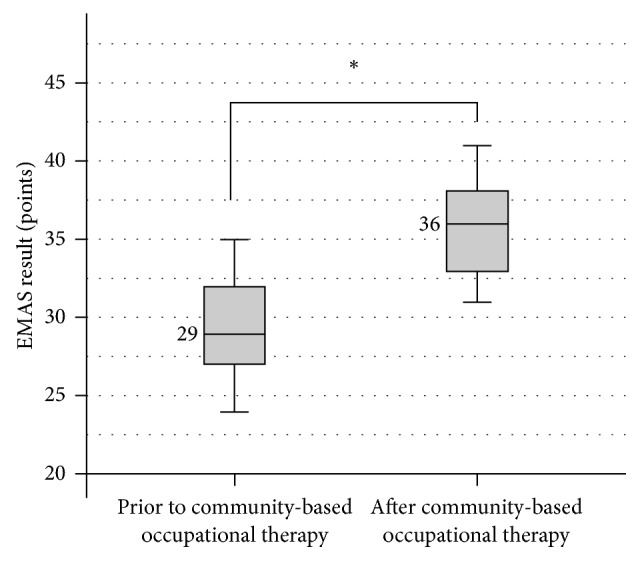
Changes in the engagement in meaningful activities in the experimental group participants, ^*∗*^*p* = 0.005.

During the evaluation of the participants' engagement in meaningful activities at baseline, the median score in the experimental group was 29 points, the lowest score being 24 points (insufficient engagement) and the highest score being 35 points (moderate engagement). At the end of the study, after the application of the 6-week community-based occupational therapy program, the evaluation scores of the participants' engagement in meaningful activities changed statistically significantly (*Z* = −2.829; *p* = 0.005): the median score was 36 points, the lowest score being 31 points (moderate engagement) and the highest score being 42 points (good engagement), *p* < 0.05 (Figure 1).

We also analyzed the relationship between breast cancer patients' engagement in meaningful activities and changes in their health-related quality of life. The results of the analysis showed that when evaluating the health-related quality of life on the EORTC QLQ-C30 questionnaire, the evaluation scores showed a statistically significant direct moderate relationship (*r*(10) = 0.663; *p* = 0.037) between engagement in meaningful activities and the score in the emotional functions scale: better results of the evaluation of the engagement in meaningful activities (i.e., a greater engagement in meaningful activities) were associated with higher evaluation scores of emotional functions, that is, better emotional functions, *p* < 0.05.

We also found a statistically significant inverse moderate relationship (*r*(10) = −0.658; *p* = 0.039) between engagement in meaningful activities and the evaluation scores in the insomnia scale: better results of the evaluation of the engagement in meaningful activities (i.e., a greater engagement in meaningful activities) were associated with lower evaluation scores of the insomnia scale, that is, a lesser degree of the manifestation of insomnia, *p* < 0.05.

We found a statistically significant inverse moderate relationship (*r*(10) = −0.681; *p* = 0.030) between engagement in meaningful activities and the evaluation scores in the constipation scale: better results of the evaluation of the engagement in meaningful activities (i.e., a greater engagement in meaningful activities) were associated with lower evaluation scores of the constipation scale, that is, a lesser degree of the manifestation of constipation, *p* < 0.05.

We did not find any statistically significant relationship for the evaluation scores of other functional or symptom scales of the quality of life questionnaire EORTC QLQ-C30 or any scale of the module EORTC QLQ-BR23,* p* ≥ 0.05 (Tables 6 and 7).

## 4. Discussion

A review of both Lithuanian and foreign literature revealed a lack of research or any kind of projects in the context of applying community-based occupational therapy for women with breast cancer, which encouraged us to conduct this study where objectives were set: to evaluate the short-term effects of community-based occupational therapy on changes in health-related quality of life among women with breast cancer and on changes in the experimental group women's engagement in meaningful activities and to analyze the associations between engagement in meaningful activities and changes in health-related quality of life.

In Lithuania, over 1.5 thousand new cases of breast cancer are diagnosed annually [7]. Improving screening programs and modern therapeutic techniques as well as increasing life expectancy of cancer patients result in an increasing relevance of the quality of life questions related to late consequences of the disease and its treatment and this is where the holistic approach and creative decisions of occupational therapists may be of assistance [11, 15, 22]. Based on this idea, in our study we analyzed aforementioned indices among women with breast cancer during the nonacute period of the disease.

In total, 21 women participated in and successfully completed the study. Of these, 10 women participated in a 6-week community-based occupational therapy program consisting of 4 group sessions (once a week, ~1.5 hours/day). The program was designed according to the guidelines provided in the only source of literature describing a study similar to ours—a project by Robinson (2015). The basis for those guidelines was a community-based occupational therapy program for women with breast cancer, aimed at maximizing their capacity for daily activities after the completion of the treatment [23].

We evaluated health-related quality of life by using the questionnaires of the European Organization for Research and Treatment of Cancer (EORTC) Quality of Life Group—the global EORTC QLQ-C30 questionnaire and its module designed specifically for breast cancer, EORTC QLQ-BR23. These questionnaires have been translated into and adapted in many languages, including Lithuanian, and are suitable for the evaluation of health-related quality of life in individuals with breast cancer [24]. In our participants, we evaluated health-related quality of life twice—at baseline and after 6 weeks. The results of our study showed that, at the beginning of the study, the women's quality of life and emotional, cognitive, and social functions were impaired; they experienced stronger manifestations of insomnia and breast and arm symptoms and were concerned about their future perspectives and the financial impact of the disease. Meanwhile, upon completion of the 6-week community-based occupational therapy program, the evaluation results of these and other aspects of health-related quality of life—such as physical functions, role functions, and fatigue—improved statistically significantly, compared to the respective results in the control group participants who did not participate in the program.

EORTC questionnaires have been recommended by a number of foreign researchers [13, 22, 25–28]. The results of a systematic survey conducted by Ghislain et al. (2016) showed that a large proportion (44%) of their analyzed studies on health-related quality of life in women with breast cancer employed EORTC questionnaires. The aforementioned survey showed that the second most frequently used (39%) instrument for the evaluation of health-related quality of life was the FACT-B* (Functional Assessment of Cancer Therapy-Breast)* questionnaire [29]. Apart from those two questionnaires, other researchers applied FACT-G* (Functional Assessment of Cancer Therapy-General)*, FACT-BSC* (Functional Assessment of Cancer Therapy-Breast Cancer Specific Subscale)*, QLACS* (Quality of Life in Adult Cancer Survivors)*, SF-36* (Medical Outcomes Short-Form Health Survey)*, and other instruments [11, 30, 31].

According to literature data, active engagement in meaningful activities positively affects the quality of life, and thus, in order to improve breast cancer patients' health-related quality of life, the women were involved in meaningful activities during the occupational therapy sessions [18, 19]. To evaluate changes in the study participants' engagement in meaningful activities and its relationship with health-related quality of life, we used the engagement in meaningful activities questionnaire during community-based occupational therapy. The women filled out this questionnaire at the beginning and at the end of the study. A survey of literature failed to identify any Lithuanian or foreign studies on the evaluation of breast cancer patients' engagement in meaningful activities. For this reason, we cannot compare our results, which showed that in the experimental group women who participated in the 6-week community-based occupational therapy program, engagement in meaningful activities improved significantly and that greater result was associated with a significant improvement in emotional functions and a reduction in the level of insomnia. We found only one study conducted by foreign researchers Pergolotti et al. (2015), where, based on the idea of participation in personally important activities for the improvement of the quality of life, the researchers analyzed participation in meaningful activities among older patients with cancer. Of the study subjects, 40% were breast cancer patients, whose participation in meaningful activities was evaluated by applying the MAPA* (The Meaningful Activity Participation Assessment)* test [32].

In this study, we strived to reveal the essence of occupational therapy in the community of women with breast cancer and the influence of this therapy on those women's health-related quality of life. The results of the study showed that occupational therapy significantly improves breast cancer patients' health-related quality of life and engagement in meaningful activities on the community level during the nonacute period of the disease.

One of the limitations of this study is a small number of participants in the groups, and thus in the future, this or a similar study should be performed with a larger sample. We also think that, in further studies, it would be expedient to evaluate whether a longer period of program application or a greater number of sessions would affect the results and improve the effectiveness of the program. In addition, future studies could be expanded by involving specialists of other areas (psychologists, art therapists, marketing specialists, etc.) because a successful implementation of the program requires not only the general competence of an occupational therapist, but also organizational skills, knowledge in marketing, and so forth in order to induce interest, attract, and invite the maximal possible numbers of women. The strong side of the study is that this was a new study in our country and that it provided positive emotions not only for the participants of the study, but also for hospitalized women who did not participate in the study, as voluntary assistance to others added meaningfulness to the sessions.

## Figures and Tables

**Table 1:** Participants' characteristics.

Characteristic	Total *n* (%)	Experimental group *n* (%)	*p* value *⟷*	Control group *n* (%)
*Age*				
Young or middle-aged (18–59 years)	11 (50)	6 (54.5)	1^*∗*^	5 (45.5)
Elderly or old (60–80 years)	11 (50)	5 (45.5)		6 (54.5)
*Employment status*				
Employed or a student	9 (40.9)	4 (36.4)	1^*∗∗*^	5 (45.5)
Unemployed, housewife, retired, or not working because of disability	13 (59.1)	7 (63.6)		6 (54.5)
*Duration of disease*				
<5 years	11 (50)	5 (45.5)	1^*∗*^	6 (54.5)
≥5 years	11 (50)	6 (54.5)		5 (45.5)
*Stage of the disease*				
0, I, or II	17 (77.3)	10 (90.9)	0.311^*∗∗*^	7 (63.6)
III or IV	5 (22.7)	1 (9.1)		4 (36.4)
*Location of the tumor*				
Left	12 (54.5)	6 (54.5)	1^*∗*^	6 (54.5)
Right	10 (45.5)	5 (45.5)		5 (45.5)
*Metastases*				
Present	3 (13.6)	1 (9.1)	1^*∗∗*^	2 (18.2)
Absent	19 (86.4)	10 (90.9)		9 (81.8)
*Functional status according to ECOG*				
0 or 1 b.	17 (77.3)	8 (72.7)	1^*∗∗*^	9 (81.8)
2 b.	5 (22.7)	(27.3)		2 (18.2)

^*∗*^Statistical significance was determined according to the *χ*^2^ criterion with Yates correction, no significant differences between the groups, *p* ≥ 0.05. ^*∗∗*^Statistical significance was determined according to Fisher's exact criterion, no significant differences between the groups, *p* >0.05.

**Table 2:** Description of the application of the occupational therapy program in the community of women with breast cancer.

	Topic	Aims	Activities
Session I	The beginning of the study	Familiarization, presentation of the study and the planned program, and initiation of the study.	Self-introduction and presentation of the study, a report on the topic of the study, initial filing out of the questionnaires, questions, and answers.

Session *II*	*“Plant and Give” *	Socializing, cooperation, increasing the hand movement amplitude or prevention, development of positive emotions, and sharing positive experience and emotions with those facing the same or similar problems.	Sapling replanting (gardening therapy), advice and discussions about the hand movement amplitude, and donation of saplings to hospitalized women with breast cancer.

Session *III*	*“Origami Crane – the Gift of Hope” *	Experiencing relaxation and calmness, expression of emotions, socialization, prevention or reduction of lymphedema, and sharing positive experience and emotions with those facing the same or similar problems.	Folding paper cranes (art therapy) + a lecture, exercises, and discussions on lymphedema treatment and prevention, as well as on an occupational therapist's assistance.

Session *IV*	*“Sharing the Strength” *	Experiencing relaxation and calmness, expression of emotions, socialization, presentation of the energy conservation principles and their practical application, and sharing positive experience and emotions with those facing the same or similar problems.	Making an original picture postcard (art therapy) while listening to specially selected calm music (music therapy) + a lecture and discussions on fatigue and energy conservation techniques (planning, adjustment of the environment, biomechanics of the body, etc.).

Session *V*	*“Knitting for Preterm Newborns” *	Gratuitous assistance.	Knitting socks + a lecture on the need for socks and donation of socks to preterm newborns.

Session VI	The end of the study	Discussion on the work done, sharing impressions and results, and conclusion of the study.	Summing-up of the program, repeated filling out of the questionnaires, feedback, discussions, questions/answers, etc.

**Table 3:** Distribution of evaluation scores in health-related quality of life questionnaire scales in the groups at baseline.

Scales of the quality of life questionnaires	Experimental group		*U*; *p* *⟷*	Control group	
	*n* (11)	*x*_me_(*x*_min_–$x_{max};\overset{-}{x})$		*n* (11)	*x*_me_(*x*_min_–$x_{max};\overset{-}{x})$
EORTC QLQ C-30					
Global health status/QoL	11	50 (33.33–83.33; 50.75)	41.5; 0.217	11	58.33 (33.33–91.67; 59.85)
*Functional scales*					
Physical functioning	11	60 (40–86.67; 63.64)	50; 0.519	11	73.33 (46.67–86.67; 67.88)
Role functioning	11	66.67 (16.67–100; 68.18)	60; 1	11	66.67 (33.33–100; 69.7)
Emotional functioning	11	50 (8.33–100; 52.27)	36.5; 0.116	11	66.67 (8.33–100; 65.91)
Cognitive functioning	11	50 (0–83.33; 54.55)	35.5; 0.101	11	83.33 (50–100; 72.73)
Social functioning	11	50 (16.67–83.33; 48.49)	32; 0.065	11	66.67 (50–100; 69.7)
*Symptom scales*					
Fatigue	11	55.56 (33.33–77.78; 54.55)	54.5; 0.699	11	44.44 (11.11–88.89; 50.5)
Nausea and vomiting	11	0 (0–50; 7.58)	54.5; 0.699	11	0 (0–66.67; 12.12)
Pain	11	33.33 (0–83.33; 34.85)	56; 0.797	11	33.33 (0–83.33; 28.79)
Dyspnoea	11	0 (0–33.33; 15.15)	50; 0.519	11	33.33 (0–66.67; 24.24)
Insomnia	11	66.67 (0–100; 66.67)	39; 0.171	11	66.67 (0–66.67; 48.49)
Appetite loss	11	0 (0–33.33; 9.09)	44; 0.3	11	33.33 (0–33.33; 18.18)
Constipation	11	33.33 (0–100; 42.42)	48; 0.438	11	0 (0–100; 24.24)
Diarrhoea	11	0 (0–66.67; 9.09)	60; 1	11	0 (0–100; 12.12)
Financial difficulties	11	66.67 (33.33–100; 69.7)	42; 0.243	11	66.67 (0–100; 51.52)

EORTC QLQ BR-23					
*Functional scales*					
Body image	11	58.33 (0–83.33; 51.51)	28.5; 0.562	11	41.57 (25–91.67; 48.48)
Sexual functioning	11	100 (0–100; 81.82)	60; 1	11	100 (33.33–100; 81.82)
Sexual enjoyment	5	66.67 (33.33–100; 73.33)	9; 0.548	5	66.67 (33.33–100; 60)
Future perspective	11	33.33 (0–66.67; 33.33)	51.5; 0.562	11	33.33 (0–66.67; 27.27)
*Symptom scales*					
Systemic therapy side effects	11	23.81 (4.76–61.90; 31.6)	58; 0.898	11	28.57 (4.76–57.14; 29)
Breast symptoms	11	41.67 (8.33–75; 38.64)	41; 0.217	11	25 (0–66.67; 28.79)
Arm symptoms	11	33.33 (0–77.78; 37.37)	63; 0.898	11	44.44 (0–88.89; 38.38)
Upset by hair loss	6	16.67 (0–33.33; 16.67)	28.5; 0.573	8	33.33 (0–100; 29.17)

Statistical significance was determined according to the nonparametric Mann–Whitney-Wilcoxon criterion, no significant differences between the groups, *p* ≥ 0.05.

**Table 4:** Characteristics of changes in the evaluation scores of the scales of the global quality of life questionnaire EORTC QLQ-C30 in the experimental and the control group women.

Scales of the quality of life questionnaire	EGr		EGr change *Z*; *p*	CGr		CGr change *Z*; *p*	Difference between the groups at the end *U*; *p*; *r*
	Before *x*_me_	After *x*_me_		Before *x*_me_	After *x*_me_		
EORTC QLQ C-30							
Global health status/QoL	50	66.67	−2.829; 0.005^*∗*^	58.33	50	−1.956; 0.05	0; 0.001^*∗∗*^; 0.85
*Functional scales*							
Physical functioning	60	70	−2.384; 0.017^*∗*^	73.33	60	−1.012; 0.311	20; 0.013^*∗∗*^; 0.54
Role functioning	66.67	75	−1.023; 0.306	66.67	66.67	−1.409; 0.159	25.5; 0.036^*∗∗*^; 0.46
Emotional functioning	50	62.5	−2.670; 0.008^*∗*^	66.67	58.33	−1.480; 0.139	8.5; 0.001^*∗∗*^; 0.72
Cognitive functioning	50	66.67	−2.060; 0.039^*∗*^	83.33	50	−2.388; 0.017^*∗*^	10; 0.001^*∗∗*^; 0.72
Social functioning	50	66.67	−1.951; 0.051	66.67	50	−2.410; 0.016^*∗*^	13; 0.002^*∗∗*^; 0.66
*Symptom scales*							
Fatigue	55.56	44.44	−2.555; 0.011^*∗*^	44.44	66.67	−2.568; 0.010^*∗*^	5; 0.001^*∗∗*^; 0.78
Nausea and vomiting	0	0	−1.089; 0.276	0	0	−1.289; 0.197	51; 0.809
Pain	33.33	16.67	−0.933; 0.351	33.33	33.33	−0.213; 0.832	41.5; 0.349
Dyspnoea	0	0	−1; 0.317	33.33	33.33	−1.382; 0.167	32.5; 0.114
Insomnia	66.67	33.33	−2.280; 0.023^*∗*^	66.67	66.67	−1.890; 0.059	19; 0.01^*∗∗*^; 0.59
Appetite loss	0	0	−0.707; 0.480	33.33	33.33	−1.633; 0.102	48; 0.654;
Constipation	33.33	16.67	−1.890; 0.059	0	0	−0.730; 0.465	48; 0.654;
Diarrhoea	0	0	−1; 0.317	0	0	−0.816; 0.414	40.5; 0.314;
Financial difficulties	66.67	66.67	−1.236; 0.216	66.67	66.67	−2.041; 0.041^*∗*^	23.5; 0.024^*∗∗*^; 0.52

^*∗*^Statistical significance was determined according to the nonparametric Wilcoxon criterion; the differences between the groups were significant when *p* < 0.05. ^*∗∗*^Statistical significance was determined according to the nonparametric Mann–Whitney-Wilcoxon criterion; the differences between the groups were significant when *p* < 0.05. *Note*. EGr: experimental group; CGr: control group.

**Table 5:** Characteristics of changes in the evaluation scores of the scales of the global quality of life questionnaire module EORTC QLQ-BR23 in the experimental and the control group women.

Scales of the quality of life questionnaire	EGr		EGr change *Z*; *p*	CGr		CGr change *Z*; *p*	Difference between the groups at the end *U*; *p*; *r*
	Before *x*_me_	After *x*_me_		Before *x*_me_	After *x*_me_		
EORTC QLQ BR-23							
*Functional scales*							
Body image	58.33	58.33	−0.512; 0.609	41.67	41.67	−1.2; 0.23	53; 0.918
Sexual functioning	100	66.67	−2.041; 0.041^*∗*^	100	83.33	0; 1	32.5; 0.114
Sexual enjoyment	66.67	50	−1.604; 0.109	66.67	66.67	−0.816; 0.414	4.5; 0.095
Future perspective	33.33	33.33	−0.184; 0.854	33.33	33.33	−0.184; 0.854	55; 1
*Symptom scales*							
Systemic therapy side effects	23.81	23.81	−2.092; 0.036	28.57	38.1	−2.371; 0.018^*∗*^	11; 0.001^*∗∗*^; 0.68
Breast symptoms	41.67	25	−2.352; 0.019^*∗*^	25	33.33	−0.511; 0.61	24.5; 0.029^*∗∗*^; 0.48
Arm symptoms	33.33	23.61	−0.986; 0.324	44.44	44.44	−0.106; 0.915	48; 0.654
Upset by hair loss	16.67	33.33	−1.633; 0.102	33.33	33.33	−1.841; 0.066	22.5; 0.852

^*∗*^Statistical significance was determined according to the nonparametric Wilcoxon criterion; the differences between the groups were significant when *p* < 0.05. ^*∗∗*^Statistical significance was determined according to the nonparametric Mann–Whitney-Wilcoxon criterion; the differences between the groups were significant when *p* < 0.05.

**Table 6:** Relationship between engagement in meaningful activities and evaluations scores of health-related quality of life (EORTC QLQ-C30) in women with breast cancer.

Scales of the quality of life questionnaire	Relationship with changes in the engagement in meaningful activities *r*(*n*); *p*
EORTC QLQ-C30	
Global health status/QoL	*r*(10) = −0.046; *p* = 0.899
*Functional scales*	
Physical functioning	*r*(10) = 0.26; *p* = 0.469
Role functioning	*r*(10) = 0.21; *p* = 0.561
Emotional functioning	*r*(10) = 0.663; *p* = 0.037^*∗*^
Cognitive functioning	*r*(10) = 0.041; *p* = 0.91
Social functioning	*r*(10) = −0.051; *p* = 0.888
*Symptom scales*	
Fatigue	*r*(10) = −0.316; *p* = 0.374
Nausea and vomiting	*r*(10) = 0.239; *p* = 0.506
Pain	*r*(10) = −0.039; *p* = 0.916
Dyspnoea	*r*(10) = 0.425; *p* = 0.221
Insomnia	*r*(10) = −0.658; *p* = 0.039^*∗*^
Appetite loss	*r*(10) = −0.619; *p* = 0.056
Constipation	*r*(10) = −0.681; *p* = 0.03^*∗*^
Diarrhoea	*r*(10) = −0.121; *p* = 0.739
Financial difficulties	*r*(10) = −0.326; *p* = 0.359

^*∗*^Statistical significance was determined according to Spearman's correlation coefficient; the association was statistically significant when *p* < 0.05.

**Table 7:** Relationship between engagement in meaningful activities and evaluations scores of health-related quality of life (EORTC QLQ- BR23) in women with breast cancer.

Scales of the quality of life questionnaire	Relationship with changes in the engagement in meaningful activities *r*(*n*); *p*
EORTC QLQ-BR23	
*Functional scales*	
Body image	*r*(10) = −0.048; *p* = 0.895
Sexual functioning	*r*(10) = −0.289; *p* = 0.418
Sexual enjoyment	*r*(10) = −0.079; *p* = 0.9
Future perspective	*r*(10) = −0.353; *p* = 0.317
*Symptom scales*	
Systemic therapy side effects	*r*(10) = 0.177; *p* = 0.624
Breast symptoms	*r*(10) = −0.423; *p* = 0.223
Arm symptoms	*r*(10) = −0.083; *p* = 0.819
Upset by hair loss	*r*(6) = 0.141; *p* = 0.79

Statistical significance was determined according to Spearman's correlation coefficient; the association was statistically significant when *p* < 0.05.
